# Assessment of the Efficiency of a ChatGPT-Based Tool, MyGenAssist, in an Industry Pharmacovigilance Department for Case Documentation: Cross-Over Study

**DOI:** 10.2196/65651

**Published:** 2025-03-10

**Authors:** Alexandre Benaïche, Ingrid Billaut-Laden, Herivelo Randriamihaja, Jean-Philippe Bertocchio

**Affiliations:** 1 Bayer Healthcare SAS France Lille France; 2 SKEZI, Les Papèteries—Image Factory Annecy France; 3 Assistance Publique—Hôpitaux de Paris, Pitié-Salpêtrière Hospital Paris France

**Keywords:** MyGenAssist, large language model, artificial intelligence, ChatGPT, pharmacovigilance, efficiency

## Abstract

**Background:**

At the end of 2023, Bayer AG launched its own internal large language model (LLM), MyGenAssist, based on ChatGPT technology to overcome data privacy concerns. It may offer the possibility to decrease their harshness and save time spent on repetitive and recurrent tasks that could then be dedicated to activities with higher added value. Although there is a current worldwide reflection on whether artificial intelligence should be integrated into pharmacovigilance, medical literature does not provide enough data concerning LLMs and their daily applications in such a setting. Here, we studied how this tool could improve the case documentation process, which is a duty for authorization holders as per European and French good vigilance practices.

**Objective:**

The aim of the study is to test whether the use of an LLM could improve the pharmacovigilance documentation process.

**Methods:**

MyGenAssist was trained to draft templates for case documentation letters meant to be sent to the reporters. Information provided within the template changes depending on the case: such data come from a table sent to the LLM. We then measured the time spent on each case for a period of 4 months (2 months before using the tool and 2 months after its implementation). A multiple linear regression model was created with the time spent on each case as the explained variable, and all parameters that could influence this time were included as explanatory variables (use of MyGenAssist, type of recipient, number of questions, and user). To test if the use of this tool impacts the process, we compared the recipients’ response rates with and without the use of MyGenAssist.

**Results:**

An average of 23.3% (95% CI 13.8%-32.8%) of time saving was made thanks to MyGenAssist (*P*<.001; adjusted *R*^2^=0.286) on each case, which could represent an average of 10.7 (SD 3.6) working days saved each year. The answer rate was not modified by the use of MyGenAssist (20/48, 42% vs 27/74, 36%; *P*=.57) whether the recipient was a physician or a patient. No significant difference was found regarding the time spent by the recipient to answer (mean 2.20, SD 3.27 days vs mean 2.65, SD 3.30 days after the last attempt of contact; *P*=.64). The implementation of MyGenAssist for this activity only required a 2-hour training session for the pharmacovigilance team.

**Conclusions:**

Our study is the first to show that a ChatGPT-based tool can improve the efficiency of a good practice activity without needing a long training session for the affected workforce. These first encouraging results could be an incentive for the implementation of LLMs in other processes.

## Introduction

Artificial intelligence (AI) is currently the center of attention in a lot of disciplines. Health is no exception. For instance, AI could be useful for the analysis of pictures for detecting local signs of catheter-associated infections [1] or for reducing both the time and costs required for drug discovery [2]. It could also play a role in the education of future health care professionals [3].

Studies from previous years already provided evidence that the use of AI would benefit pharmaceutical companies [4-6]. However, most of those studies used complex applications of AI to extract or synthesize data from documents like health records [7,8] or social media content [9-11].

Although not yet reachable to a wide audience, large language models (LLMs) offer possibilities to integrate AI in several domains without requiring a long training of the workforce, thanks to its accessibility and ease of use. While everyone can use some LLMs that are open access—including ChatGPT since November 2022 [12]—getting an adequate answer sometimes requires gradually improving the prompts. Hence, the integration of LLMs in the work environment can provide new soft skills at a low cost and promote the empowerment of workers. Several studies already showed that pharmacists and other health care professionals are willing to use LLMs as a help in their work [13-15].

However, the use of LLMs raises concerns about data privacy. Recent news showed that the information provided by some users in their prompts could be transferred by ChatGPT to others [16]. This threat can be answered by companies with the opportunity to develop their own LLM for internal use.

Hence, Bayer AG launched on September 21, 2023, its own internal LLM called MyGenAssist, based on ChatGPT technology. Bayer, as a market authorization holder in Europe, has the duty to set a system to collect, register, and analyze adverse events related to its products according to European [17] and French good pharmacovigilance practices (GVPs) [18] and the European Directive 2001/83/CE [19]. Tasks related to this imperative follow internal procedures, which make some of them repetitive and time-consuming. A semiautomatization of these tasks thanks to an LLM could make them less arduous for workers and procure more time for other activities at higher added value.

In the pharmacovigilance field, the Giens Workshop [20] in 2022 aimed to initiate a reflection on the integration of AI in this area and highlighted the actions implying the writing of a letter to contact a patient or a health care professional as a good opportunity for this. To the best of our knowledge, no study described LLMs’ use cases in pharmacovigilance from a daily and practical perspective.

In this study, we aim to determine whether the use of MyGenAssist in the pharmacovigilance case documentation process can provide an improvement in efficiency (ie, save time without leading to a decrease in the answer rate).

## Methods

### Description of the Activity and Materials Used

#### Description of the Case Documentation Process and Potential Contribution of MyGenAssist

**Figure 1:**
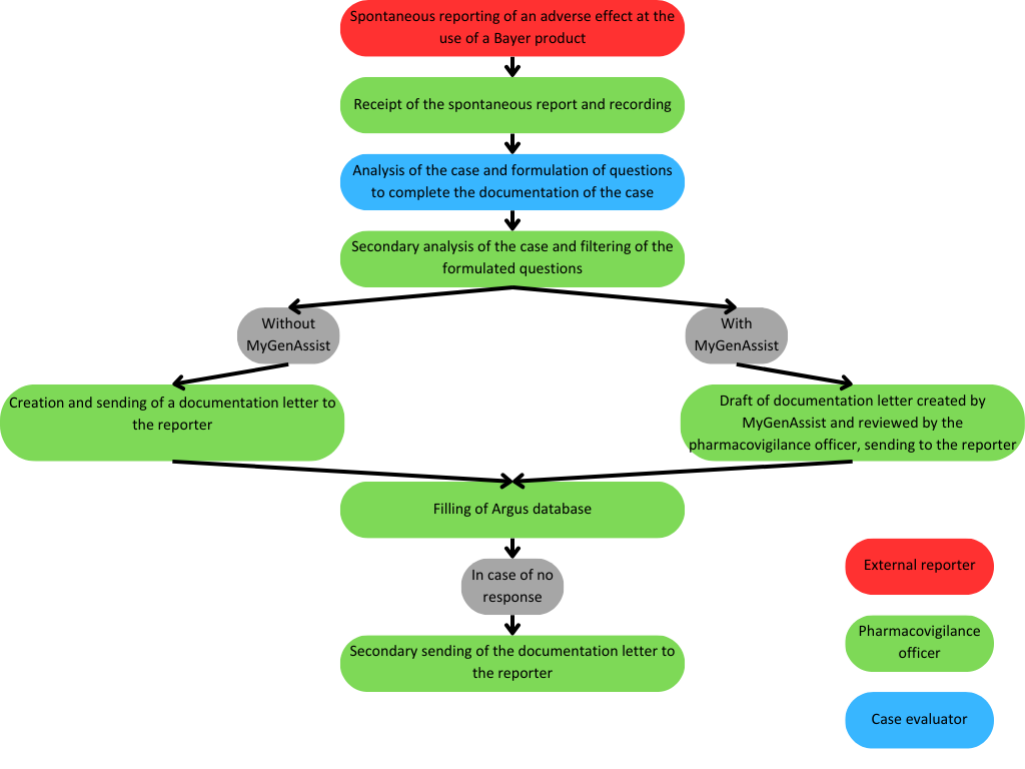
Description of the process for documenting spontaneous notifications of adverse drug events.

The current internal process for collecting, recording, and documenting spontaneous case reports of adverse drug events at Bayer France is described here (Figure 1): reporters can notify Bayer about cases by phone, mail, or electronic means. The report is compiled into a source document, which is then added to an internal pharmacovigilance database by a local pharmacovigilance officer. The source document is analyzed by a case evaluator who is part of the company’s global pharmacovigilance team. Based on the global analysis, the pharmacovigilance officer is in charge of defining the final list of relevant questions to complete the case. The GVPs, written by the French Agence Nationale de Sécurité du Médicament et des produits de santé, add a duty to contact the reporter a second time if the first attempt of contact was unsuccessful [18].

MyGenAssist was added to this process at the stage of drafting documentation letters. This LLM was used to write a first draft of the letters, which was then reviewed and modified if needed by the pharmacovigilance officer.

#### Software and Applications Used

MyGenAssist is an LLM internal to Bayer based on the technology of ChatGPT-4 Turbo (OpenAI), a conversational agent based on generative AI [21]. The model used here has no differences compared to the one available to the public, apart from the fact that MyGenAssist is securely hosted by Bayer. All manipulations performed on this internal LLM can be reproduced on ChatGPT, which is accessible to the public on the web. Here, MyGenAssist was used by ourselves as final users; the prompt was created without any fine-tuning or modification of its algorithm.

Pharmacovigilance cases reported to Bayer are compiled on an internal pharmacovigilance database supported by the Argus pharmacovigilance case management software developed by Oracle [22]. This software assigns a specific reference to each case. It contains all documents related to the case as well as information regarding the actions taken to analyze the case, such as attempts to contact the reporter.

The questions formulated by the case evaluators following their analysis of the case are listed on the FAST application (Bayer internal application). The pharmacovigilance officer can select the relevant questions and add others if needed.

#### Use of MyGenAssist for the Activity

Pharmacovigilance case documentation letters were written according to templates validated by an internal process. Two templates exist depending on the type of recipient (patients or health care professionals). The pharmacovigilance officer could adapt the template in function of the pharmacovigilance case. To make the LLM predraft the pharmacovigilance case documentation letters, the 2 templates were used. The elements of the templates corresponding to information specific to each case were replaced by titles placed into brackets in the letter templates. These 2 letter templates were then provided via the following prompt as shown in Multimedia Appendix 1 (original version in French; the translation corresponds to Multimedia Appendix 2, an example of a query is shown in Multimedia Appendix 3, and its translation is shown in Multimedia Appendix 4).

### Staff Training

To train staff to use MyGenAssist specifically for this activity, a training session was planned before initiating its use. The earlier-described operating mode was given to the pharmacovigilance officers. A training phase was also planned to verify their good understanding of the use of the tool. The operating procedure was also formalized for the use of potential new arrivals. To allow pharmacovigilance officers to report any difficulties or suggestions for improvement regarding the operating procedure, meetings were scheduled every other week until the termination of the study.

### Data Collection

The study was conducted between January 2 and May 3, 2024. The time spent on each case was measured from the moment the pharmacovigilance case was acknowledged until the letter was sent and the Argus database was filled out.

Over an initial period of 9 weeks, the time spent on drafting each documentation letter, without the use of MyGenAssist, was measured. Over a second equivalent period, the time spent on the same task, but using the LLM as a drafting aid, was also measured. During the 2 periods, apart from the addition of MyGenAssist, no significant changes in the management of the activity took place: 3 workers, including 2 experimented participants and a newcomer were present throughout the 4 months of the study, and the process did not undergo any notable modifications.

### Statistical Analysis

#### Calculation of Time Savings

**Figure 2:**
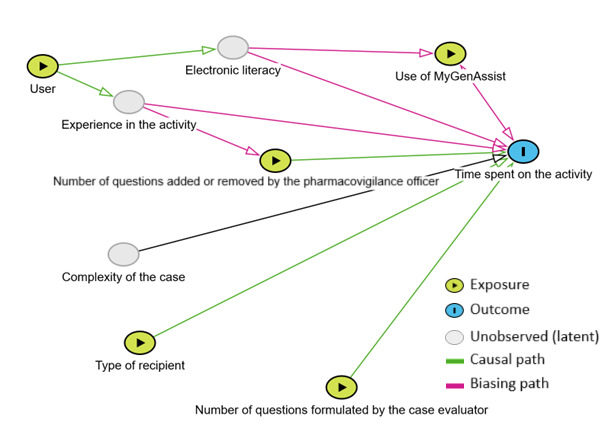
Causal diagram of factors potentially impacting the time spent on the activity, built with Dagitty software (version 3.1).

To determine the average time saved for each case thanks to MyGenAssist, a multiple linear regression was performed, in which the explained variable or outcome was the time spent per case. To highlight the impact of MyGenAssist, the parameters that could potentially influence the time spent per case were researched and selected on the basis of a review of the pharmacovigilance documentation process with experienced pharmacovigilance officers who defined, based on their daily practice, which elements could weigh on the time spent. Because of the company’s specificities in the pharmacovigilance documentation process, the judgment of pharmacovigilance officers was then the unique criteria to add these parameters to the model. Among them, all parameters that could be modelized were included in the regression model as independent or explanatory variables (Figure 2):

*Y*=*a*_1_*X*_1_+*a*_2_*X*_2_+*a*_3_*X*_3_+*a*_4_*X*_4_+*a*_5_*X*_5_+*b*

*Y* is the time spent per case (continuous quantitative variable, expressed in minutes). *X*_1_ is the use of MyGenAssist (dichotomous categorical variable: use or no use). *X*_2_ is the type of recipient (dichotomous categorical variable: patient or health care professional). The letter template differs significantly depending on this parameter. It may also be necessary to simplify the questions contained in the letter for patients, leading to more time spent on the task. *X*_3_ is the number of questions formulated by the case evaluator (discrete quantitative variable). This parameter can also impact the time spent. Each question is asked in English. Without the use of MyGenAssist, a French translation is automatically provided by the internal software FAST, but the translations need to be reviewed and the questions sometimes need to be adapted according to the recipient. This adaptation is a step in the workflow whose duration could be correlated to the number of questions. When using MyGenAssist, this latter also provides a translation of the questions, but some adaptation work is still necessary. *X*_4_ is the number of questions added or removed by the pharmacovigilance officer (discrete quantitative variable). The pharmacovigilance officer has the option to remove questions prepared by the case evaluator considering them irrelevant in the context of the case. Conversely, this latter can also add questions to obtain information considered as necessary. Adding questions or considering the removal of questions from the case processor leads to additional reflection time that can impact the overall duration of the task. *X*_5_ is the user (categorical variable: user 1, 2, or 3). The user was taken into account. The personal characteristics of the user will influence the time spent on the task. *a*_1_, *a*_2_, *a*_3_, *a*_4_, and *a*_5_ are, respectively, the coefficients of the variables *X*_1_, *X*_2_, *X*_3_, *X*_4_, and *X*_5_, and *b* is the intercept. Three parameters in this causal diagram were not included in the model because they cannot be measured: the experience in the activity, the electronic literacy of the user, and the complexity of the case.

The multiple linear regression was performed using the R software (R Foundation for Statistical Computing) with the “lm” function, which can be found in the *stats* package (all R equations used in this study are described in Multimedia Appendix 5). The outliers were researched thanks to the function “check_outliers” from the *performance* package [23].

The linear regression was completed with a hierarchical regression to study the relevance of the independent variables in the model. It was performed with the function “stepAIC” from the package *mass* [24] on R. The hierarchical regression is a backward stepwise Akaike information criterion.

Cases were randomly distributed among the pharmacovigilance officers without considering the context of the case, the type of recipient, and the number of questions a priori. The number of questions formulated was not related to the type of recipient.

#### Measurement of Effectiveness

The reliability of the LLM was not studied because the use of MyGenAssist was intended only as an aid in drafting documentation letters. However, a measure of the effectiveness of the task with or without the tool was carried out. For this, a comparison of the proportion of cases for which a response from the recipient was obtained after the first and the second contact attempt was made, depending on whether MyGenAssist was used or not. A subgroup analysis was also realized in function of the type of recipient (physician or patient). The comparison was made on R thanks to the “chisq.test” function that can be found in the *stats* package. In the subgroup analysis, the same comparison was done but specifically for cases whose recipient was a physician on one hand, and on the other hand, for cases whose recipient was a patient.

### Ethical Considerations

This study was not considered as being related to human research in health, as it clearly focused on evaluating work performance with or without an AI-based tool. Regulations in France require such studies to be in compliance with the General Data Protection Regulation (ie, European Regulation 2016/679) [25]. The data used in this study were retrieved first during the normal course of the pharmacovigilance activities and reused for the study as authorized by General Data Protection Regulation [25] and after having received oral consent from pharmacovigilance workers who were informed of the objective of the study and how the data will be used.

## Results

### Statistical Study

**Figure 3:**
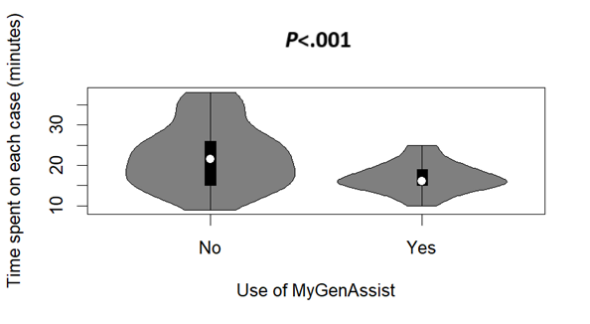
Time spent on each case in function of the use of MyGenAssist.

**Figure 4:**
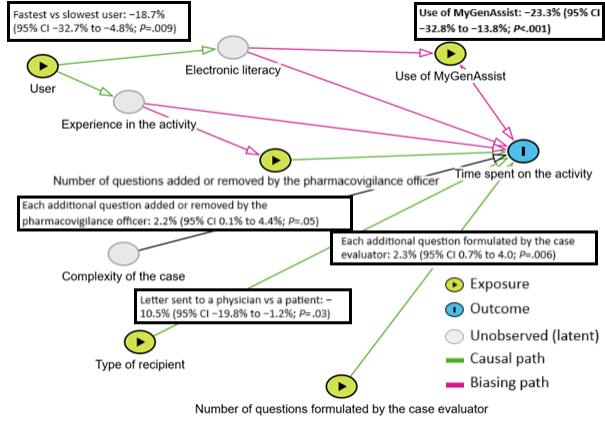
Influence of explanatory variables on the time spent on a case. A logarithmic transformation was performed to fulfill the conditions of the linear regression model (the actual model is log *t*=a1X1+a2X2+a3X3+a4X4+a5X5+b). “log T” is the logarithmic transformation of the variable T; hence, the result obtained is a percentage of difference of time in function of each parameter.

The study included 122 cases (48 without the use of MyGenAssist and 74 with its use). The average time spent on each case was 19.05 (SD 6.05; 95% CI 17.97-20.12) minutes. The average time for cases handled was 22.25 (SD 7.62; 95% CI 20.09-24.41) minutes without the use of MyGenAssist and 16.97 (SD 3.50; 95% CI 16.17-17.77) minutes when the LLM was used (Figure 3). When all factors that potentially impact the time spent on the activity are taken into account in a multiple linear regression model, an average of 23.3% (95% CI 13.8%-32.8%; *P*<.001) of time savings were realized for each case thanks to MyGenAssist (adjusted *R*^2^=0.286; *df*=115; Figure 4). All explanatory variables of the linear regression model got a *P* value inferior to .05 apart from the number of questions added or removed by the pharmacovigilance officer (*P*=.05).

The hierarchical regression performed found that all the parameters used as explanatory variables were useful to get the best model (the lowest Akaike information criterion is obtained with all the parameters included in the model; Table 1).

**Table 1:** Akaike information criterion (AIC) of the multilinear regression model in function of the parameters used.

Parameters included	*df*	Sum of squares	Residual sum of squares	AIC
All	—^a^	—	7.0929	–333.08
Variable “number of questions added or removed by the pharmacovigilance officer” removed	1	0.23076	7.3237	–331.17
Variable “type of recipient” removed	1	0.30945	7.4024	–329.87
Variable “user” removed	2	0.44116	7.5341	–329.72
Variable “number of questions formulated by the case evaluator” removed	1	0.48239	7.5753	–327.05
Variable “use of MyGenAssist” removed	1	1.45757	8.5505	–312.28

^a^Not applicable.

According to the number of spontaneous pharmacovigilance reports for which a documentation request was necessary during the last 3 years, it can be estimated that the mean time saved on the activity with the use of the LLM could be between 66.27 (SD 22.79) and 108.70 (SD 37.23) hours per year, on the basis of an average 23.3% time saved on the average 22.25 (SD 7.62) minutes spent on a case without the use of MyGenAssist. Based on a working day of 8 hours, the use of MyGenAssist for this activity in the 3 previous years could have saved an average of 10.7 (SD 3.6) days of work per year (Table 2).

**Table 2:** Potential time saved on the pharmacovigilance case documentation process each year with the use of MyGenAssist.

Year	Pharmacovigilance cases requiring documentation request, n	Potential time saved (hours)		Potential time saved (working days)^a^	
		Mean (SD)	95% CI	Mean (SD)	95% CI
2021	1258	108.70 (37.23)	98.16-119.23	13.6 (4.7)	12.3-14.9
2022	947	81.82 (28.02)	73.89-89.75	10.2 (3.5)	9.2-11.2
2023	767	66.27 (22.79)	60.12-72.72	8.3 (2.8)	7.5-9.1

^a^A working day is considered to last 8 hours.

No statistical difference has been found regarding the answers’ rates after 1 attempt of contact, whether MyGenAssist was used or not: 31% (15/48) of letters produced without MyGenAssist were provided with an answer and 24% (18/74) when the generative AI was used for the writing (*P*=.40; *χ*^2^_1_=0.7). The same situation occurred after 2 attempts: 42% (20/48) without the tool vs 36% (27/74; *P*=.57; *χ*^2^_1_=0.3). In a subgroup analysis in function of the type of recipient, these results were consistent (Table 3).

**Table 3:** Rates of answers to letters sent for pharmacovigilance case documentation in function of the use of MyGenAssist and the type of recipient.

	Without the use of MyGenAssist, n/N (%)	With the use of MyGenAssist, n/N (%)	*P* value	Chi-square (*df*=1)
Total	20/48 (42)	27/74 (36)	.57	0.3
Physicians	14/29 (48)	15/45 (33)	.20	1.6
Patients	6/19 (32)	12/29 (41)	.49	0.5

**Figure 5:**
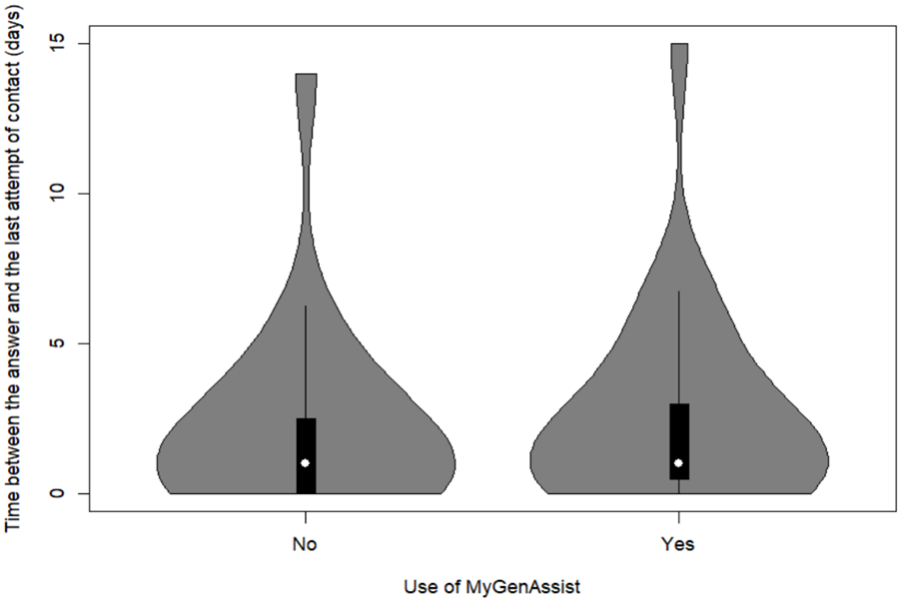
Comparison of the duration time between the answer and the last attempt of contact in function of the use of MyGenAssist.

In cases for which an answer is provided, the average time between the answer and the last attempt of contact does not differ significantly (Figure 5): 2.20 (SD 3.27; 95% CI 1.27-3.13) days when the letter was written without MyGenAssist and 2.65 (SD 3.30; 95% CI 1.90-3.41) days with its use (*P*=.64; t_44_=–0.46).

### Staff Meetings

Before the study, a 2-hour training was planned to present the use of MyGenAssist in the pharmacovigilance case documentation process to the pharmacovigilance officers. During the study, meetings were planned in the team on a 2-week basis. No difficulties in the use of MyGenAssist were identified, and users only suggested slight improvements in the prompt used to consider specific cases for which additional elements are required in the letter. An operating mode, detailing all the steps required to use MyGenAssist for the activity, was written to offer a reminder for the users if necessary.

## Discussion

### Main Results

In this study, we demonstrated that the implementation of MyGenAssist to the pharmacovigilance case documentation process provided an average of 23.3% time saving on the task without any modification in the rate of answers. Whatever the experience of the workers, the use of MyGenAssist induces time savings, although this effect is higher in nonexperimented ones. Moreover, while using MyGenAssist, it seems that the time spent on the different pharmacovigilance cases becomes less variable: when the use of the tool is added to the process, the range of time is decreased from 9-38 minutes to 10-25 minutes. MyGenAssist may have a more beneficial impact in cases for which more time is required. The easiest cases needing a slight amount of time to be handled would benefit less (or not) from its use, but the ones that are the more time-consuming would be realized significantly quicker than without it.

Therefore, the use of Bayer’s LLM improved the efficiency of this activity. That must save dozens of hours yearly for our local pharmacovigilance team, which could be dedicated to other activities with higher added value. A unique, short, and reachable training was sufficient to implement the use of MyGenAssist. This consisted of introducing the changes in the tool’s operating mode to the users. No additional training content was required to enable them to use the LLM in the activity, though they did not have any specific qualifications or skills in informatics. Hence, we showed in this study that making workers use an LLM could be easy, as far as they are assisted at the beginning, while other AI tools require important training to be used daily. However, the daily use of an LLM can make the user continually improve their abilities with this tool. Therefore, LLMs seem easy to integrate into several work environments and a way for the worker to get new skills without needing a large amount of time. Thanks to its ease, all users kept using the generative AI for their activity after the end of the study.

Although we included in the linear regression model all parameters that could have an influence on the time spent on the activity, in our point of view, the adjusted *R*^2^ is low (0.286). Therefore, the most part of the time spent on each case could not be fully explained by the considered parameters. It seems that some aspects that are specific to each and difficult to transcribe in the model are important factors too. Moreover, a “human factor” could play a role here, as the user could spend a variable duration of time for the same task, in function of a lot of exterior parameters. Whatever explanatory variable was not included here, the average difference created by MyGenAssist (more than 5 minutes on a task requiring about 22 minutes without its use) seems too high to be questioned by these potential “hidden” parameters.

Nonetheless, 3 factors could not be included in the model. First, the experience of the user in the activity may directly influence the time spent on each case. It could also have an effect on the number of questions added or removed by the pharmacovigilance officer: while a newcomer could have difficulties selecting the required information to request from the recipient, an experienced one would more accurately determine which question is important enough to assess an adverse event. Second, electronic literacy, which is the capacity to understand or create messages using electronic means, may have an influence on the time spent on the activity. This impact exists for all cases because they are handled by using informatics, but must be stronger in the cases for which MyGenAssist would be used, as the ability of the user to handle technology could also impact their ease of familiarizing themselves with this new tool. By the time we started this experiment, we were not able to measure either user experience or user electronic literacy [26]. Even if we could not include them in the model, the variable “user” integrates both their experience and their electronic literacy: so, without deciphering which parameter has more impact, we still take them into account. Third, the complexity of the case, which may have an influence on the time spent on the case, is a subjective parameter that could not be measured accurately. The parameters we chose to include in this study were selected during a meeting with experienced pharmacovigilance officers, who were asked to give all factors that could influence the time spent on each case. It seems that no study so far, to the best of our knowledge, has been published reporting the parameters influencing the time spent to document a pharmacovigilance case. Moreover, no comparison of our internal process and the ones of other pharmaceutical companies could be done, as none of them seems to be available publicly. The European GVPs [17] do not define a strict process for the documentation of pharmacovigilance cases and let the market authorization holders organize the activity. Pharmaceutical companies have to create a Pharmacovigilance System Master File that describes the handling of all pharmacovigilance activities. It has to be sent to the national and European health authorities but is not publicly disclosed [27]. Our process could then have specificities (eg, the implication of both a case evaluator and a pharmacovigilance officer) that are not possible to detect and could create a lack of reproducibility of our study.

Other ways could have been explored to determine the effectiveness of generative AI. In this study, we chose to use the fulfillment of the objective of our task as the main criteria of effectiveness: to get the necessary information to analyze the pharmacovigilance case. From our perspective, other criteria did not seem relevant in this context. Moreover, we made the choice not to analyze the quality of the drafts written by MyGenAssist because it was clear in our operating mode and in the training that a human assessment of the letter was mandatory before sending it out. To get a relevant analysis of the effectiveness, recipients were not informed that the letter they received was first written with the help of an LLM: on the one hand, the recipient could have had the impression that its report was “automatically” handled, which could have encouraged this latter not to answer. On the other hand, the potential curiosity created by an eventual mention of generative AI, in a current era in which all eyes are turned to such tools, could have created a bias by making the recipient more willing to answer.

### Limitations

While our study was realized over a 4-month period, this latter only includes 122 cases. Even if it was enough to notice a difference in the time spent in function of the use of the tool, this has to be taken into account, particularly while analyzing the answers’ rate. However, this study is a first step before the extension of the use of the LLM in the pharmacovigilance case documentation process to all Bayer local pharmacovigilance teams worldwide. The efficiency improvement noticed by the French pharmacovigilance team could give fresh impetus to make other teams adopt the same principle. This will give us the opportunity to retrieve more data concerning our question.

### Comparison to Prior Work

Other publications regard the use of LLMs in pharmacovigilance: the use of ChatGPT without any optimization or fine-tuning of the public does not seem to fulfill the objective fixed, whatever this latter is providing answers to pharmacovigilance-related questions [28,29] or assess the risk of mortality of patients with toxic epidermal necrolysis [30]. On the other hand, another LLM, Bidirectional Encoder Representations from Transformers, was used with success for the extraction of adverse events from notes [31,32] or social media [33], but it always implied a step of fine-tuning that could not be handled by a simple user. Therefore, we could not find in the literature any study describing the successful use of ChatGPT or a strict equivalent in pharmacovigilance without the necessity to fine-tune the LLM after its use.

Moreover, the need to explore the possibilities that AI could offer to pharmacovigilance was highlighted by several publications before, particularly with the idea that the implementation of such tools could make both time and cost savings and provide an efficiency improvement, especially as the number of declarations of adverse events has been sharply increasing in the last few years [34]. Gholap et al [35] and Danysz et al [36] evoke the possibility for pharmacovigilance workers to focus on high-level tasks thanks to these time savings on administrative or repetitive tasks. However, in all these papers, these statements remain at the step of hypothesis without any experimental assessment.

Regarding the potential benefits that AI could represent for pharmacovigilance, the TransCelerate initiative, which includes 19 pharmaceutical companies, aimed to ask pharmacovigilance representatives of these companies which steps of the pharmacovigilance case processing might be the best suited for a pharmacovigilance implementation. In 2 surveys set between 2018 [37] and 2021 [38], the pharmacovigilance workers considered the pharmacovigilance case documentation process as one of the more appropriate steps for an AI implementation. The automation opportunity score, which combines the effort realized by companies to include AI in the task and the benefit or risk assessment regarding this inclusion, was 4.5/5 in 2021. The percentage of companies that set an AI-based process in this task rose from 19% to 35% between 2020 and 2021. Nonetheless, no information was given in these publications about a possible efficiency increase after an AI implementation.

Hence, our study may fulfill what seems to be, to the best of our knowledge, a lack in the medical literature. Descriptions of LLMs’ use cases for pharmacovigilance exist, but none of them include a measure of time saving realized thanks to this tool. A study showed that the use of ChatGPT for writing tasks enhanced the productivity of the workers by decreasing the time required by 40%, while the quality rose by 18% [39], but did not concern health. Hence, our objective was to determine if the integration of the LLM in the adverse event case documentation process can improve efficiency by reducing the time required for this GVP-related task without decreasing the response rate from the reporters.

These first encouraging results could be an incentive to implement MyGenAssist in other processes. In the pharmacovigilance field, some tasks fit well with the integration of MyGenAssist. For example, the comparison of different versions of the same procedure with this tool can provide a rapid insight into the modifications in order in a second step to assess their impact on other quality documents. Other use cases could be found in other departments, as the task given to the LLM, which is writing letters based on a template, seems pretty reproducible in other contexts for different objectives. The results of this study show that using LLMs in pharmaceutical activities, whatever the field, is relevant and can create improvements without losing quality concerning the regulations of good practice activities.

### Conclusions

In this study, we showed the first example of a use case for a ChatGPT-based tool, MyGenAssist, in a pharmacovigilance industry department and assessed its efficiency over a 4-month period. An average of 23.3% of time savings was achieved thanks to this LLM, while its implementation did not modify the effectiveness of the task. It only required a short training to be set up. These results could be the first step to the largest use of LLMs in pharmaceutical activities.

## Appendix

Multimedia Appendix 1 Original prompt used in French.

Multimedia Appendix 2 Translation of the prompt used in English.

Multimedia Appendix 3 Example of a query by the user and output generated by MyGenAssist in French.

Multimedia Appendix 4 Translation in English of an example of a query by the user and output generated by MyGenAssist.

Multimedia Appendix 5 Description of the R equations used in the paper.

## Abbreviations

AI: artificial intelligence
GVP: good pharmacovigilance practices
LLM: large language model

## Data Availability

The datasets generated or analyzed during this study are not publicly available due to our internal data privacy policies, but specific elements are available from the corresponding author on reasonable request.
