# Supplementary figures and images for: Assessment of the Efficiency of a ChatGPT-Based Tool, MyGenAssist, in an Industry Pharmacovigilance Department for Case Documentation: Cross-Over Study
Source: J Med Internet Res. 2025 Mar 10;27:e65651. doi: 10.2196/65651 (PMC11933758; doi:10.2196/65651)

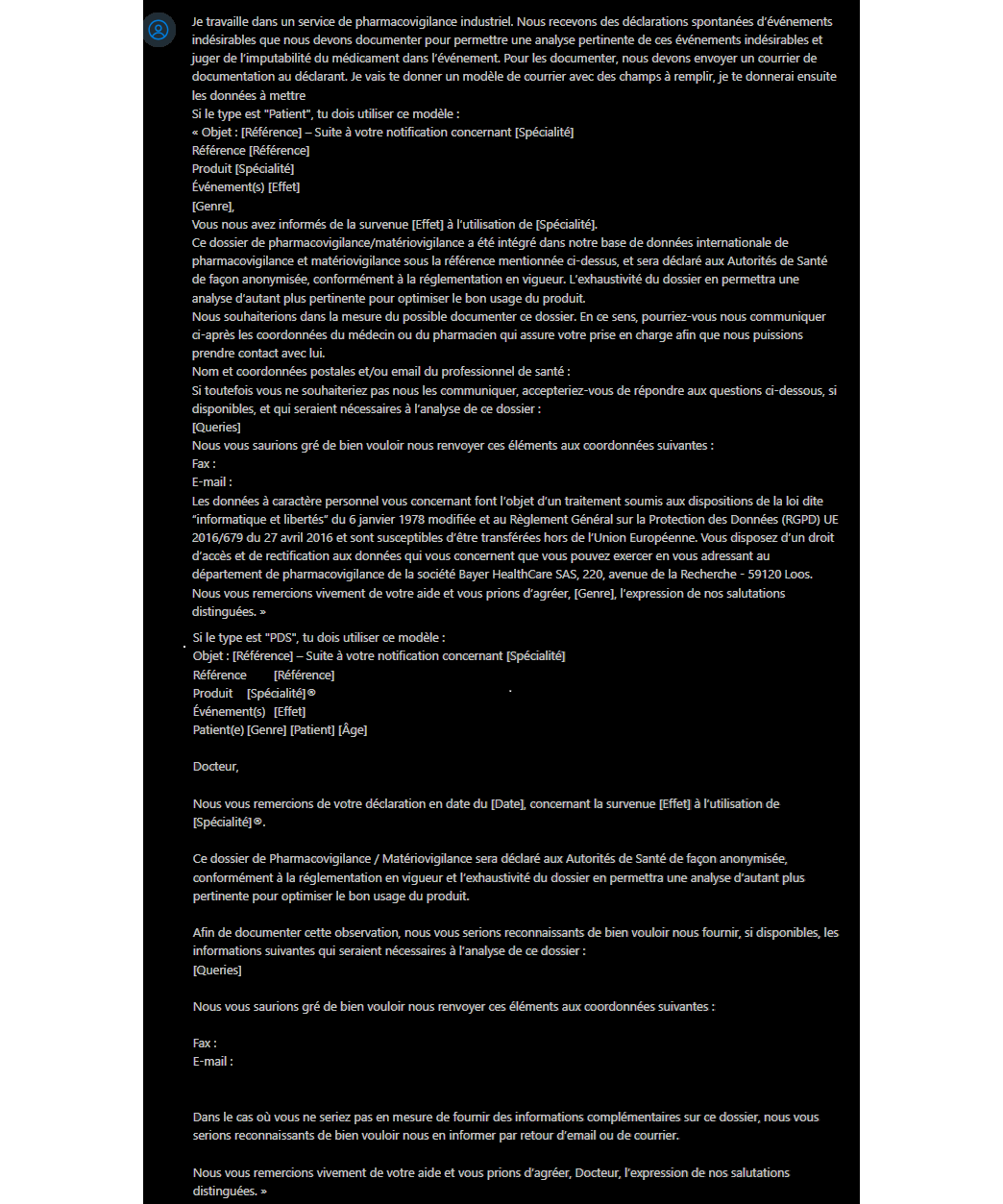

Supplement: Multimedia Appendix 1 [file jmir_v27i1e65651_app1.png]

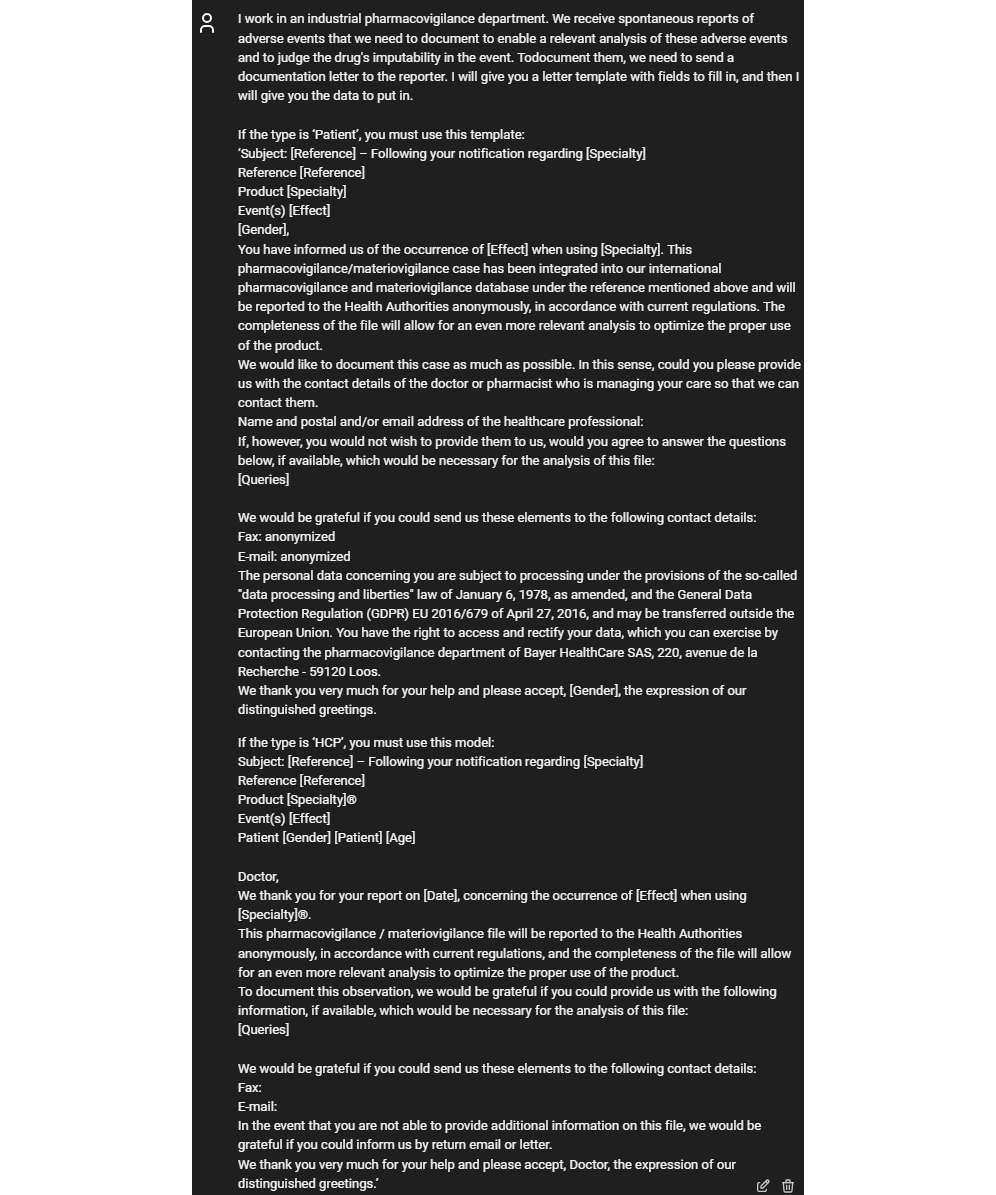

Supplement: Multimedia Appendix 2 [file jmir_v27i1e65651_app2.png]

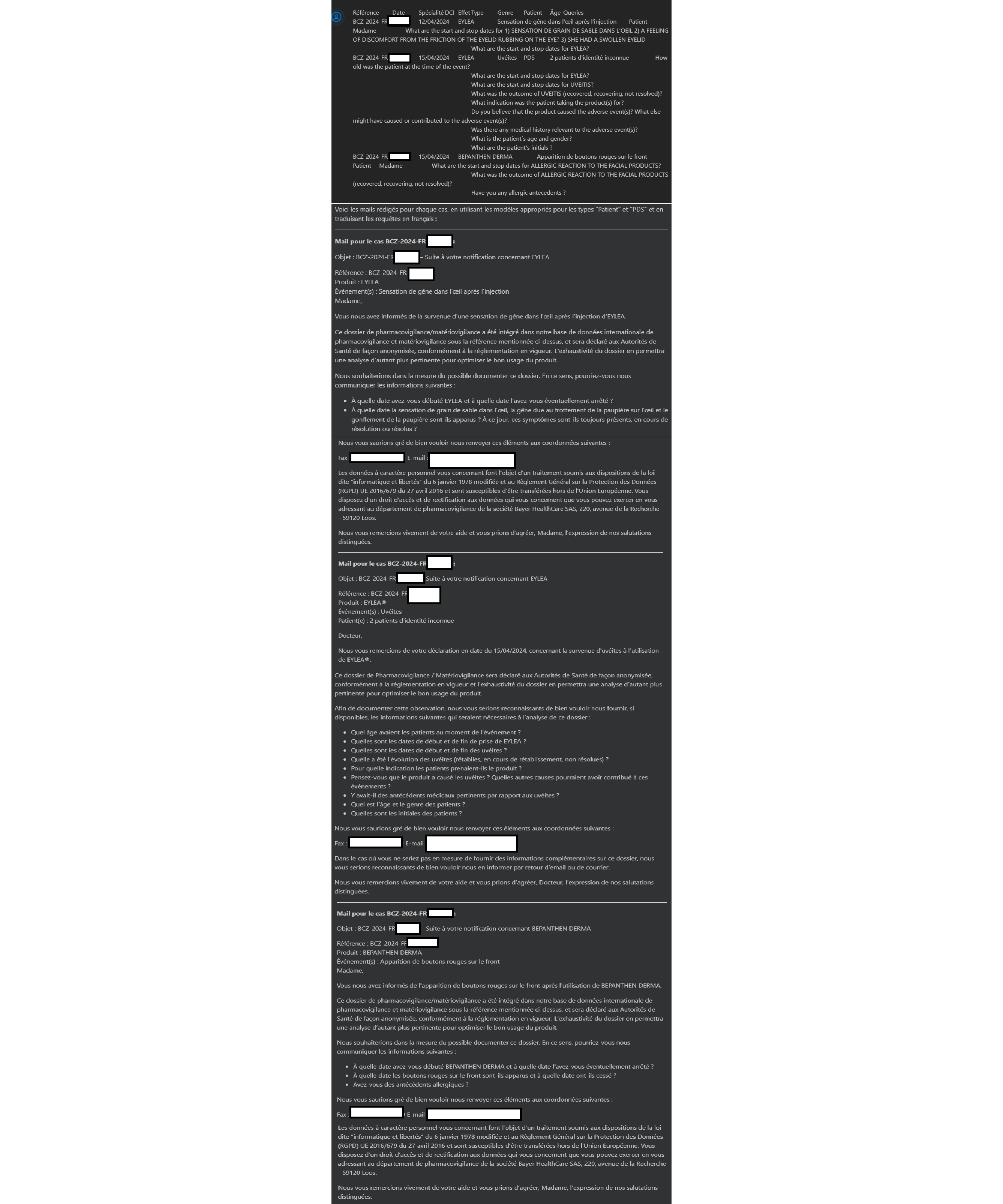

Supplement: Multimedia Appendix 3 [file jmir_v27i1e65651_app3.png]

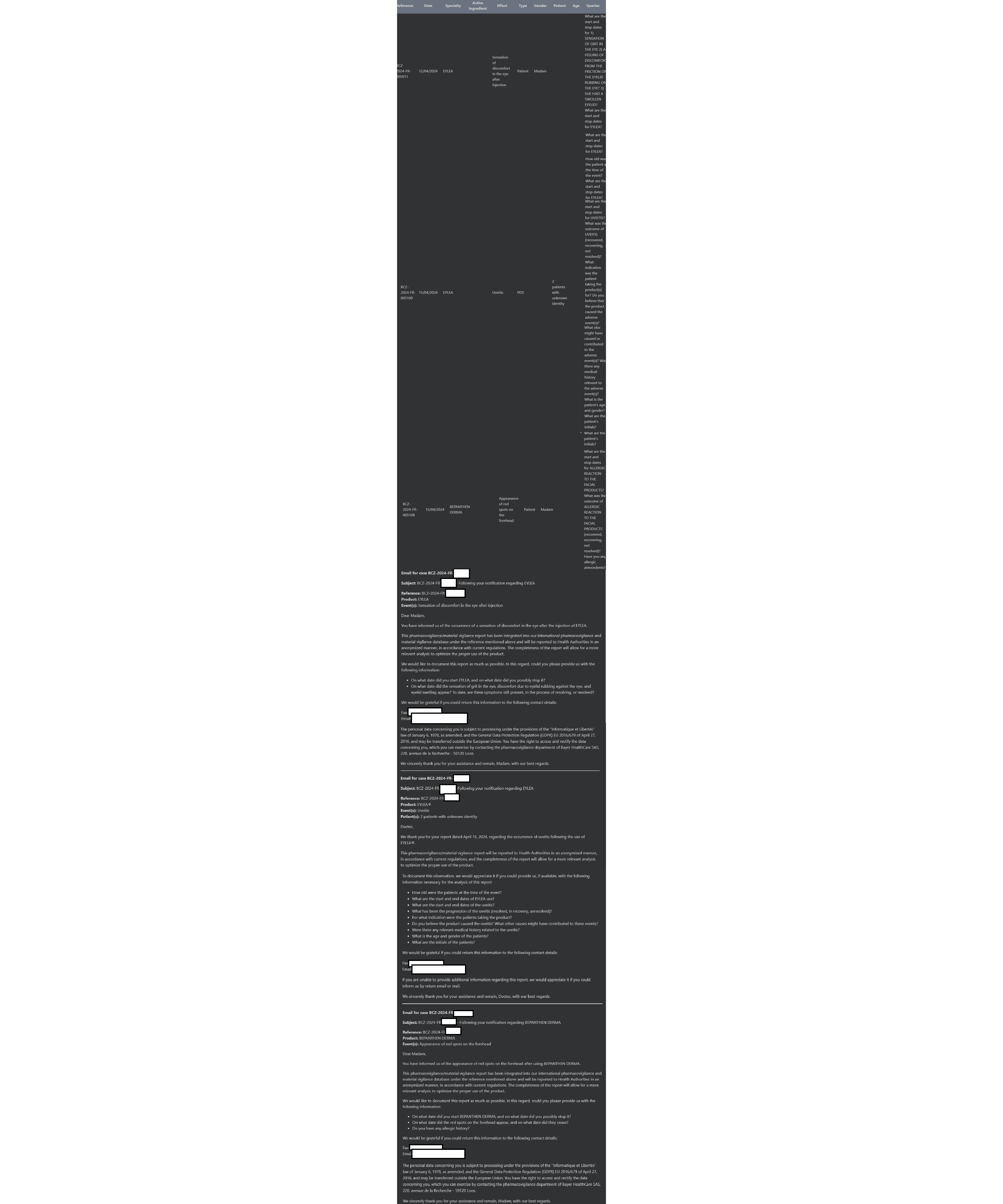

Supplement: Multimedia Appendix 4 [file jmir_v27i1e65651_app4.png]
