# Supplementary material for: Assessment of the Efficiency of a ChatGPT-Based Tool, MyGenAssist, in an Industry Pharmacovigilance Department for Case Documentation: Cross-Over Study
Source: J Med Internet Res. 2025 Mar 10;27:e65651. doi: 10.2196/65651 (PMC11933758; doi:10.2196/65651)
Supplement: Multimedia Appendix 5 [file jmir_v27i1e65651_app5.docx]

| **Statistical analysis realized** | **Section of the article concerned** | **R^®^ function used** | **R^®^ package used** | **Equation** | **Explanation on the equation writing** |
| --- | --- | --- | --- | --- | --- |
| Linear regression model regarding the time spent on each case | Methods  Statistical analysis  Calculation of time savings | lm | stats | Model<-lm(T~D+G+A+MGA+U,data=Queries) | Model: name given to the multiple linear regression model.  T: time spent in minutes.  D: type of recipient (patient or physician, ‘destinataire’ in French, coded 0 for a patient, or 1 for a physician).  G: number of questions firstly formulated by the case evaluator.  A: number of questions secondly added or removed by the PV officer.  MGA: use of MyGenAssist^®^ (coded 0 if the tool wasn’t used for the case, 1 otherwise).  U: user (coded 1, 2 or 3 as there were 3 users who participated in this study). |
| Verification of the absence of outliers in the dataset used to create the linear regression model |  | check_outliers | performance | check_outliers(Model) | This function searches for outliers in the dataset (‘Model’). No outliers were found here. |
| Hierarchical regression (backward stepwise Akaike Information Criterion) to study the relevance of the independent variables |  | stepAIC | mass | hierarchical_regression <- stepAIC(Model, trace = TRUE, direction = "both") | Model: name given to the multiple linear regression model.  trace = TRUE: instruction to R to display the values in the console.  direction = ‘both’: instruction to R to create the more adequate model between a forward or a backward stepwise. Here, we got a backward stepwise. |
| Comparison of the proportion of answers obtained in function of the use of MyGenAssist^®^ (khi-2 test). | Methods  Statistical analysis  Measurement of effectiveness | chisq.test | stats | chisq.test(Queries$R2,Queries$MGA,correct=FALSE) | R2: answer (coded 0 if no answer was obtained, 1 otherwise).  MGA: use of MyGenAssist^®^ (coded 0 if not used, 1 if used). |
| Logarithmic transformation of the linear regression model | Results  Statistical study | lm | stats | Model<-lm(Tlog~D+G+A+MGA+U,data=Queries) | The equation of the linear regression model had to be modified to fulfil the conditions  Tlog: corresponds to log(T). |
